# Supplementary material for: Seismic hazard analysis and financial impact assessment of railway infrastructure in the US West Coast: A machine learning approach
Source: PLoS One. 2024 Aug 12;19(8):e0308255. doi: 10.1371/journal.pone.0308255 (PMC11318867; doi:10.1371/journal.pone.0308255)
Supplement: S2 File — A comprehensive dataset detailing earthquakes in California, Oregon, and Washington from 2000 to 2022 with magnitudes of 3.5 or greater. It includes annual statistics such as the number of earthquakes per state, minimum, mean, and maximum depths, and magnitudes recorded. This data supports the study on seismic hazards and their impact on railway infrastructure along the U.S. West Coast. Data sources include the U.S. Geological Survey (USGS). (PDF) [file pone.0308255.s002.pdf]

Supplementary Information

Data Availability

This study examines the seismic hazard impact on railway infrastructure along the U.S. West Coast (Washington, Oregon, and California). To support the findings, we provide access to the datasets used in our analysis. These datasets include measures of seismic hazard such as fault density, earthquake frequency, and ground shaking, as well as the recorded railway infrastructure accidents from 2000-2022.

Downloadable Data Links

1. Seismic Hazard Data:
   a. Quaternary Faults:https://www.usgs.gov/natural-hazards/earthquake-hazards/faults
   b. Seismic Hazard Map for the United States: http://purl.stanford.edu/rm034qp5477
2. Railway Infrastructure Accidents Data: https://safetydata.fra.dot.gov/officeofsafety/publicsite/Query/inccaus.aspx.
3. Railway Network Location Shapefile: https://data.ca.gov/dataset/california-rail-network
4. US State and County Boundary Shapefiles:
   a. CA Geographic Boundaries: https://data.ca.gov/dataset/ca-geographic-boundaries
   b. US State and County Boundaries: https://www.census.gov/geographies/mapping-files/time-series/geo/carto-boundary-file.html

Supplementary File

We also provide a CSV file containing the earthquakes data downloaded from (U.S. Geological Survey, n.d.). We also further calculate overall statistics of the earthquake data as follows:

Year	State	Earthquakes	Min. Depth (km)	Mean Depth (km)	Max. Depth (km)	Min. Magnitude	Mean Magnitude	Max. Magnitude
**2000**	California	76	0.0	6.38	25.06	3.5	3.89	4.9
**2000**	Oregon	2	0.0	0.0	0.0	3.6	3.85	4.1
**2000**	Washington	3	6.7	21.22	49.56	3.5	3.57	3.6
**2001**	California	68	0.0	5.98	22.0	3.5	3.97	5.2
**2001**	Washington	5	3.9	31.59	52.31	3.9	4.8	6.8
**2002**	California	49	0.89	8.4	22.94	3.52	3.85	4.9
**2002**	Oregon	3	4.92	5.39	6.31	3.8	4.2	4.5
**2002**	Washington	1	15.73	15.73	15.73	3.7	3.7	3.7
**2003**	California	123	0.0	6.17	26.7	3.5	3.87	6.5
**2003**	Oregon	1	17.09	17.09	17.09	3.9	3.9	3.9
**2003**	Washington	3	19.55	31.62	50.51	3.51	4.0	4.8

**2004**	California	100	0.0	6.33	31.47	3.5	3.91	5.97
**2004**	Oregon	6	0.0	6.09	10.42	3.5	3.85	4.4
**2004**	Washington	20	0.0	3.37	55.47	3.5	3.63	4.01
**2005**	California	78	0.0	7.82	34.64	3.5	3.99	5.2
**2005**	Oregon	1	5.0	5.0	5.0	3.8	3.8	3.8
**2005**	Washington	3	0.0	7.49	13.19	3.5	3.67	4.0
**2006**	California	61	0.0	7.19	27.66	3.5	3.87	4.71
**2006**	Washington	6	0.0	2.65	14.25	3.5	3.77	4.5
**2007**	California	52	0.0	7.67	25.98	3.5	3.95	5.45
**2007**	Oregon	3	17.65	21.28	23.43	3.6	3.67	3.8
**2007**	Washington	2	46.67	49.63	52.6	3.57	3.71	3.85
**2008**	California	59	0.0	7.18	27.76	3.5	3.92	5.44
**2008**	Oregon	3	15.49	16.48	17.59	3.6	3.8	4.2
**2008**	Washington	1	19.61	19.61	19.61	3.7	3.7	3.7
**2009**	California	84	0.0	5.62	20.26	3.5	3.91	5.19
**2009**	Oregon	2	15.33	26.06	36.8	3.6	3.92	4.24
**2009**	Washington	3	43.64	54.75	62.24	3.6	4.07	4.67
**2010**	California	173	0.0	6.76	30.76	3.5	3.86	5.71
**2010**	Oregon	2	14.47	15.23	16.0	3.6	3.6	3.6
**2010**	Washington	3	1.28	5.99	14.07	3.8	4.07	4.2
**2011**	California	50	0.0	7.44	25.4	3.52	3.87	4.73
**2011**	Washington	5	1.6	21.87	51.71	3.63	4.02	4.6
**2012**	California	78	0.48	8.97	30.32	3.5	3.97	5.6
**2012**	Oregon	1	23.81	23.81	23.81	3.54	3.54	3.54
**2012**	Washington	1	7.75	7.75	7.75	3.57	3.57	3.57
**2013**	California	59	0.0	6.69	33.54	3.5	3.86	5.69
**2013**	Washington	4	6.45	10.24	13.75	3.6	3.81	4.27
**2014**	California	58	0.0	6.08	14.65	3.5	3.87	6.02
**2014**	Oregon	1	4.3	4.3	4.3	3.83	3.83	3.83
**2014**	Washington	2	26.41	42.24	58.06	3.5	3.51	3.53
**2015**	California	45	0.0	7.37	21.05	3.51	3.79	4.43
**2015**	Oregon	2	7.95	13.98	20.01	3.51	3.82	4.14
**2015**	Washington	4	3.56	10.41	18.83	3.58	3.95	4.2
**2016**	California	59	0.0	8.41	35.62	3.5	3.97	5.19
**2016**	Washington	1	35.24	35.24	35.24	3.5	3.5	3.5
**2017**	California	50	0.0	8.58	34.83	3.5	3.75	4.58
**2017**	Oregon	1	17.37	17.37	17.37	3.96	3.96	3.96
**2017**	Washington	3	15.44	38.01	56.45	3.58	3.79	4.09

**2018**	California	48	0.0	6.55	16.33	3.51	3.8	4.5
**2018**	Washington	2	10.23	24.78	39.32	3.87	3.97	4.07
**2019**	California	467	0.0	6.15	25.83	3.5	3.86	7.1
**2019**	Oregon	3	16.13	25.6	44.02	3.54	3.88	4.53
**2019**	Washington	1	28.82	28.82	28.82	4.58	4.58	4.58
**2020**	California	139	0.0	7.3	28.6	3.5	3.87	5.8
**2020**	Oregon	2	8.45	27.98	47.5	3.52	3.52	3.53
**2021**	California	128	0.0	7.71	33.89	3.5	3.89	6.2
**2021**	Oregon	1	4.3	4.3	4.3	3.94	3.94	3.94
**2021**	Washington	1	43.84	43.84	43.84	3.78	3.78	3.78
**2022**	California	67	0.0	10.93	33.39	3.51	3.88	5.06
**2022**	Oregon	1	13.17	13.17	13.17	4.39	4.39	4.39
**2022**	Washington	2	13.54	18.43	23.33	3.56	3.64	3.71
