# Supplementary material for: Seismic hazard analysis and financial impact assessment of railway infrastructure in the US West Coast: A machine learning approach
Source: PLoS One. 2024 Aug 12;19(8):e0308255. doi: 10.1371/journal.pone.0308255 (PMC11318867; doi:10.1371/journal.pone.0308255)
Supplement: Attachment — Submitted filename: [PONE-D-24-16061] - Response to Reviewers.pdf [file pone.0308255.s003.pdf]

Responses to reviewers

Reviewer #1: The authors of the paper touched upon a very important topic. On the one hand, they determined the relationship between the frequency of earthquakes and railroad accidents, and on the other hand, they linked these data to the costs of railroad infrastructure.
However, the methods used by the authors can be called classical. It would be interesting to use modern machine learning methods. It is unclear why the authors, for example, did not use deep neural networks. In my opinion, for publication in a journal of this level, the authors should have conducted a deeper analysis in at least one of the two selected areas.

Thank you for your insightful comments on our paper. We appreciate your recognition of the importance of the topic and the relationships we have established between the frequency of earthquakes, railroad accidents, and the costs of railroad infrastructure.

Regarding your suggestion to use modern machine learning methods such as deep neural networks, we considered this approach. However, we opted for classical methods primarily due to the interpretability they offer. Our goal was not only to predict outcomes but also to understand how individual features contribute to the model and their relationships with the target variables. Deep neural networks, while powerful, often operate as "black boxes," making it difficult to interpret and explain the influence of individual features on the model's predictions.

We believe that the transparency provided by classical methods is crucial for stakeholders and decision-makers who rely on our findings for practical applications and policy-making. Nonetheless, we acknowledge the potential of advanced machine learning techniques and consider them a valuable direction for future research to complement our current findings.

To address this in the manuscript, we also added, “We chose the classical machine learning models primarily for their interpretability. Our aim is not only to predict outcomes but also to understand how individual features contribute to the model and their relationships with the target variables” in 2.2 Modeling section (lines 283).

Comments
1. The manuscript can be divided into two parts. The first part, devoted to establishing the connection between earthquakes and railroad accidents, is technically competent. Classical methods are used, the literacy of which is not in doubt. As for the second part - proposals for the development of railroad infrastructure - I cannot give an assessment.

Thank you for your positive comments.

2. I can't spin off the dataset, so I can't check the validity of the statistical analysis.

Thank you for pointing this out. We are now providing our earthquake data retrieved from USGS in a csv format, Quaternary Faults and Seismic Hazard map in shapefiles formats. The links to download all seismic-related data are also included in the Reference list.

3. The authors did not provide full access to all data underlying the results of their manuscript.

As mentioned previously, we now include Supplementary datasets with the manuscript.

4. The manuscript is written in clear language.

Thank you for your kind words. We

are pleased to hear that you found the manuscript clearly written.

Reviewer #2: The manuscript should provide more detailed descriptions of the datasets used. While it mentions the sources (Federal Railroad database and U.S. Geological Survey database), it should include details such as the number of data points from each, time periods covered, and any preprocessing steps taken to refine the inputs to the model.

Thank you for raising this issue. We previously mentioned in section (1) Earthquakes (lines 213) that our earthquake data are those occurring from 2000 to 2022. However, we did not provide the information on the number of data points. Thus, we currently added more detailes on the earthquake data, "The total number of earthquakes in this dataset is 2,282 events. We only used the earthquakes that had magnitude greater than 3.5 because earthquakes starting from this magnitude can produce seismic waves that can be detected globally by seismographs, offering clearer and more reliable data (Kuang et al., 2021). This facilitates the precise determination of earthquake parameters, including magnitude, location, and depth. Additionally, these earthquakes are more likely to cause noticeable surface effects, such as structural damage and felt ground shaking (Münchmeyer et al., 2021)." (lines 223).

For the preprocessing, we also mentioned in the same section, "Since there is a wide range of both hypocenter depths and magnitudes of earthquakes (see Supplementary Information for additional details on the dataset), we divided the events based on these two factors. We then determined the number of earthquakes occurring annually in each county, using the following classification:

- EQ-MC1: Magnitude ≥ M3.5 earthquakes, all depths,

- EQ-MC2: Magnitude ≥ M4 earthquakes, all depths,
- EQ-MC3: Magnitude ≥ M5 earthquakes, all depths,
- EQ-HD1: Magnitude ≥ M3.5 earthquakes and hypocenter depths ≤ 10 km,
- EQ-HD2: Magnitude ≥ M3.5 earthquakes and hypocenter depths ≤ 20 km,
- EQ-HD3: Magnitude ≥ M3.5 earthquakes and hypocenter depths ≤ 40 km." (lines 234).

We also included the earthquake data (csv file), active faults and seismic hazard (ground shaking motion) shapefiles in the Supplementary Information, and we also provided the URL to download the railway infrastructure accident data.

In terms of experiment set-up, the methodology section should elaborate on control measures - for example, if there were **any control variables and/or assumptions** made during model training/testing, these should be explicitly stated. Further, **while the document captures the split of data into training and validation sets, explaining the approach used** (e.g., proportions, randomization methods) would drive transparency. Further, **information number of repeated experiments to arrive at the results is also missing. For example, specifying cross-validation techniques/detailing the number of folds will improve the result reliability**.

Thank you for your comments. Our methodology section (2.2.3 Modeling) now explicitly states the control measures, such as the use of standardized data and the assumption of normality in residuals, and details the randomization and cross-validation techniques used to ensure the reliability of our results: "We assumed that the residuals of the models follow a normal distribution, validated by examining the residual plots and conducting normality tests. It was also assumed that the observations are independent of each other, a crucial assumption for the validity of the statistical tests used. All data were standardized to have a mean of zero and a standard deviation of one, and missing values were imputed using the mean imputation method." (lines 392-396). Also, "The dataset was randomly split into training (80%) and testing (20%) sets to ensure that model performance was evaluated on unseen data. This randomization was performed using a fixed random seed to ensure reproducibility. We used 5-fold cross-validation during the training phase to optimize hyperparameters and prevent overfitting. The choice of 5-fold cross-validation was based on balancing computational efficiency with robust performance evaluation." (lines 399-403).

While the manuscript compares different models, it should also provide statistical significance of tests across these models. Including confidence intervals or related metrics will help here.

Thank you for your insightful suggestion. To address this, we further performed the statistical significance tests and confidence intervals for the RMSE values of the different models. We added Table 3 to show our results:

Table 3. Statistical significance tests relative to Linear Regression model and confidence Intervals for RMSE values of different models.

**Model**	**Confidence Intervals**	**t-statistics**	**p-values**

Linear	(14.98, 16.10)	-	-
Ridge	(7.44, 8.04)	25.13	7.25e-33
Lasso	(7.44, 8.04)	25.12	7.40e-33
Elastic Net	(7.44, 8.04)	25.12	7.30e-33
Decision Tree	(5.91, 6.43)	31.01	8.45e-38
Random Forest	(5.66, 6.19)	31.81	2.10e-38

We also added the paragraph to explain these additional findings, "Further statistical analysis confirms the significance of these findings (Table 3). Paired t-tests between the linear model and each alternative model revealed extremely low p-values (all well below 0.05), indicating that the differences in RMSE values are statistically significant. For example, the t-statistic for the comparison between the linear model and the Random Forest model was 31.81, with a p-value of approximately 2.10e-38. These results underscore the superior performance of the non-linear models. Moreover, the 95% confidence intervals for the RMSE values of the alternative models (e.g., 5.66 to 6.19 for the Random Forest) further demonstrate the precision and reliability of these models. This comprehensive evaluation highlights the robustness of our findings and the effectiveness of using RMSE for model comparison, affirming that more sophisticated models like Random Forests provide significantly improved predictive accuracy over the simple linear model." (lines 473-483)

The details around monte-carlo simulation are also missing - detailing the number of simulations and the distribution of input variables is needed.

We added the suggested details as follows "The basis for this analysis is a comprehensive data set spanning from 2000 to 2022, incorporating all counties within California, Oregon, and Washington. A Monte Carlo simulation was conducted to enhance the robustness of the sensitivity analysis. The distribution parameters are calibrated with a conservative lower bound set at 10% below the established standard cost, considering factors such as a small number of earthquakes, and an optimistic upper limit set at 60% above the standard cost, accounting for factors such as a large number of earthquakes, high inflation rates, etc. This simulation involved 5,000 iterations to ensure a comprehensive exploration of possible outcomes. The input variable for the simulation was accident cost, including time-sensitive values. This input variable was modeled using a triangular distribution…" (lines 667 - 677)

Clarification is needed on metrics used for performance evaluation and their suitability for this research - for instance the rationale behind using R-squared, Mean Absolute Error (MAE), and Root Mean Squared Error (RMSE) for model evaluation.

Thank you for your insightful comments regarding the performance evaluation metrics used in our research. We appreciate the opportunity to clarify our choice of RMSE as the sole metric for model evaluation.

In our manuscript, we chose to use Root Mean Squared Error (RMSE) for the following reasons:

- Sensitivity to Large Errors: RMSE is particularly sensitive to large errors, as it squares the deviations before averaging them. This sensitivity makes it a more stringent metric for performance evaluation, ensuring that models that make significant errors are appropriately penalized. In the context of our study, accurately predicting extreme values (such as large-scale railroad accidents due to major earthquakes) is crucial, and RMSE helps in highlighting these significant deviations.
- Reflecting Overall Model Performance: RMSE provides a comprehensive measure of the model's prediction accuracy by considering the magnitude of the error. By focusing on the square root of the average squared differences between predicted and actual values, RMSE offers a clear indication of how well the model performs across all data points, not just the average performance.
- Standard Unit of Measurement: RMSE is expressed in the same units as the dependent variable, making it easier to interpret the results. This allows us to directly understand the scale of prediction errors in the context of railroad accidents, facilitating more meaningful assessments and comparisons.
- Balancing Model Complexity and Interpretability: While other metrics like Mean Absolute Error (MAE) and R-squared are also valuable, we opted for RMSE due to its ability to balance the need for a robust evaluation metric with the complexity of the model. RMSE's sensitivity to larger errors provides a more nuanced view of model performance, especially in scenarios where large errors can have significant real-world implications.

By using RMSE as our primary evaluation metric, we ensure that our models are rigorously assessed, particularly with respect to their ability to handle extreme values and large deviations. This focus aligns with the goals of our research, where the accuracy of predictions in critical scenarios is paramount.

To address this in the manuscript, we added, “We chose RMSE as our model validation metric because it is particularly sensitive to large errors, squaring the deviations before averaging them. This sensitivity makes RMSE a stringent metric, ensuring that models with significant

errors are appropriately penalized (Hodson, 2022). Given our study's focus on accurately predicting extreme values, such as large-scale railroad accidents due to major earthquakes, RMSE is essential for highlighting these deviations. Additionally, RMSE provides a comprehensive measure of prediction accuracy by considering the magnitude of errors, offering a clear indication of model performance across all data points (Hodson, 2022). Expressed in the same units as the dependent variable, RMSE simplifies interpretation, helping us understand the scale of prediction errors in the context of railroad accidents and infrastructure costs. While other metrics, such as Mean Absolute Error (MAE) and R-squared are valuable, we opted for RMSE due to its robust evaluation capabilities and its ability to handle the complexity of our models. RMSE's sensitivity to larger errors provides a nuanced view of model performance, especially in scenarios where significant errors can have substantial real-world implications." (lines 437-450).

Other general suggestions:
Please provide the definition or citation to technical terms - for instance peak ground acceleration is not defined when it is used first. Similarly, there is lack of consistency in usage of technical terms -seismic variables and earthquake variables are used interchangeably which can create confusion to the reader

Peak ground acceleration (PGA) is first defined on Line 253 where it is first used. We have edited the manuscript to ensure that there is consistency in the usage of technical terms including seismic versus earthquake variables, seismic hazard versus seismic, and PGA.
